# Osmoregulation and the human mycobiome

**DOI:** 10.3389/fmicb.2014.00167

**Published:** 2014-04-17

**Authors:** Abhishek Saxena, Ramakrishnan Sitaraman

**Affiliations:** Department of Biotechnology, TERI UniversityNew Delhi, India

**Keywords:** mycobiome, microbiota, osmoregulation, MAP kinase, *Candida* spp., stress response, *S. cerevisiae*

## Introduction

The last one-and-a half decades have made it amply clear that the human microbiota have a very significant role to play in health and disease. The human body can (or should) be better viewed as a complex ecosystem inhabited by micro-organisms that outnumber human cells 10 to 1 (Ley et al., 2006). However, most research in this field has been focused on the prokaryotic (specifically, bacterial) component of the microbiota. Sampling, in turn, is carried out mostly from sites that are readily accessible (Human Microbiome Project Consortium, 2012). Such sampling might not be genuinely representative of the situation *in vivo*, even for the bacteria under study. The human alimentary tract is a complex entity that exhibits extensive variation in biological characteristics such as tissue/cell types and secretions, as well as parameters such as temperature, pH, oxygen levels and osmolarity along its entire length. A proteomic study sampling mucosal lavages at multiple colonic sites indicated significant differences in protein profiles between the proximal and distal colon, which was supportive of the concept of their functional and developmental distinctness (Li et al., 2011). The colonization of the human infant by microbes, initially during the process of birth, exhibits an ecological succession of microbial species over time, and plays a prominent role in the maturation of the immune system as well (reviewed in Costello et al., 2012).

The fungal members of the microbiota are not very numerous compared to bacteria. Large-scale metagenomic sequencing of fecal samples of 124 individuals found that only about 0.1% of genes detected were of eukaryotic origin (Qin et al., 2010). The most commonly encountered genera constituting the fungal microbiota or “mycobiome” (Huffnagle and Noverr, 2013) are *Candida*, *Saccharomyces* and *Cladosporium* (Hoffmann et al., 2013). The bacterial microbiota and a functional immune system are thought to keep the numbers of opportunistic fungal pathogens, such as *Candida* spp., under check in the absence of any perturbations. However, information from studies of polymicrobial diseases points to subtler adjustments, dependent on environmental conditions and cross-kingdom signals that eventually influence (positively and negatively) modes and rates of growth (reviewed in Peleg et al., 2010). The sensing of, and responses to, biotic and abiotic stimuli by fungi (as in other organisms) involves multiple signaling pathways that can interact to either augment or attenuate one another, as will be discussed below.

## Osmoregulation and stress responses in *saccharomyces cerevisiae*

*S. cerevisiae*, a well-studied fungus, employs two strategies for responding to stress, both involving extensive signaling by MAP kinases (MAPK, alternatively termed SAPK-stress activated protein kinase). The first is stress-specific, e.g., involved in the response to pheromone, spore wall formation and adapting to hyperosmotic conditions etc. (Gustin et al., 1998). The HOG (high osmorality glycerol) pathway that is activated in response to hyperosmotic conditions leads to the accumulation of compatible solutes (glycerol being the most important) and also results in the closure of the aquaglyceroporin Fps1p, enabling retention of glycerol. The HOG pathway functions through two signaling branches. The SLN1 branch involves the two-component membrane sensor protein Sln1p complexed with Ypd1p and Ssk1p that, under hyperosmotic conditions, is unable to inactivate downstream MAPKKKs (functionally redundant Ssk2p and Ssk22p) by phosphorylation. This results in the dephosphorylation of these kinases that phosphorylate the MAPKK Pbs2p which, in turn, phsophorylates the MAPK Hog1p. Phosphorylated Hog1p moves into the nucleus and interacts with transcription factors such as Hot1p, Msn1p, Msn2p etc. activating the transcription of, among other genes, including those encoding phosphatases (e.g., Ptp2p, Ptp3p) that dephosphosphorylate Hog1p, causing feedback inhibition, limiting the duration of Hog1p activity. The SHO1 branch involves two functionally redundant mucin-like transmembrane osmosensors, Msb2p and Hkr1p, that recruit the Pbs2p MAPKK directly to the cytoplasmic face of the cell membrane as part of a macromolecular complex. Notably, SHO1 branch proteins are shared with other signaling pathways, and it is activated when hyperosmolarity occurs as a result of other cellular responses (reviewed in Hohmann, 2002; Hohmann et al., 2007).

The second strategy for coping with stress is the environmental stress response (ESR) that enables adaptation to the long-term effects of various stresses, in contrast to the more specific and short-term response of other MAPK pathways. The ESR was first described as an increased expression of ~300 genes and repression of ~600 genes in response to diverse environmental conditions to which *S. cerevisiae* was subjected (Gasch et al., 2000; Causton et al., 2001). In both these studies, various stress conditions were tested including temperature, hyper- and hypo-osmotic shock, oxidative (H_2_O_2_) stress etc. Induced genes included those involved in a wide variety of processes, including carbohydrate metabolism, detoxification of reactive oxygen species, cellular redox reactions, cell wall modification, protein folding and degradation, DNA damage repair, fatty acid metabolism, metabolite transport, vacuolar and mitochondrial functions, autophagy, and intracellular signaling (Gasch et al., 2000). Genes encoding cytoplasmic ribosomal proteins, tRNA synthases, proteins required for processing rRNAs, and a subset of translation initiation factors were repressed (Causton et al., 2001).

The ESR provides a “cross-protective effect” wherein *S. cerevisiae* subjected to mild heat stress as the primary stress becomes adapted to higher levels of heat as well as oxidative (H_2_O_2_) stresses (Berry et al., 2011). This is especially relevant as stresses under natural conditions don't occur singly or sequentially, but simultaneously. It may seem at first sight that the mild dosage of primary/initial stress is irrelevant, as the ultimate adaptation to the secondary stress(es) is achieved anyway. However, Berry et al. (2011) demonstrated that distinct subsets of genes were activated due to primary and secondary stresses. Earlier work indicated that the cross-protective effect is not universal, but specific to the primary/secondary stress combination (Berry and Gasch, 2008). The major transcription factors mediating the ESR are Msn2p and Msn4p (Berry et al., 2011). Msn2p and Msn4p (see below) play specific roles depending on the stress combination and are even regulated in a condition-specific manner. Besides, there are other transcription factors activated during the ESR and subsequent “acquired stress resistance,” like Hsf1p (heat stress), Yap1p (oxidative stress), and Hot1p & Sko1p (hyperosmotic stress), that can also activate Msn2p/4p target genes (Berry and Gasch, 2008).

Adding to the mechanistic complexity of stress responses is recent evidence that Hog1p induces transcription of a long non-coding RNA whose presence is required for chromosome remodeling around the *CDC28* gene encoding a cyclin-dependent kinase and its subsequent induction. This is accompanied by cell cycle delay, and increased Cdc28p levels ensure faster recovery following the stress application (Nadal-Ribelles et al., 2014). Osmoadapated *S. cerevisiae* exhibit HOG activation upon shmooing in response to pheromone (Baltanás et al., 2013). Hog1p also imposes checkpoints on the mating pathway if pheromone is sensed during a period of hyperosmotic stress. It phosphorylates the protein kinase Rck2p that inhibits translation of Fus3p (the MAPK of mating pathway) by phosphorylating EF2 (elongation factor 2). Ste50p, a shared component of both the HOG and mating pathways is phosphorylated (and inhibited) by Hog1p (Nagiec and Dohlman, 2012). Thus, the osmoregulation response is not elicited solely by hyperosmolarity *per se*, but is also influenced by the spatio-temporal modulation of cell-cycle events involving stimuli impinging on other signaling pathways.

### The stress responses and adaptive potential of *Candida* spp.

*Candida* spp*.* are dimorphic yeasts that occur practically throughout the alimentary tract, and *C. albicans* and *C. glabrata* well-known opportunistic pathogens. *C. albicans* can grow as a planktonic unicellular organism (yeast) or as a filamentous organism (hypha). The yeast form is suitable for dissemination, while the hyphal form is more adapted to colonization and biofilm formation. *Candida albicans* also exhibits parasexuality, in which mating-competent (so-called “opaque”) diploid strains mate to form tetraploids, whose progeny later undergo chromosome losses to regenerate the diploid state (Hull et al., 2000; Magee and Magee, 2000). More recent work has demonstrated the existence of a viable and stable haploid form generated by chromosome losses implying that *C. albicans* may not be an “obligate diploid” as originally thought (Hickman et al., 2013). This increases the overall repertoire of genetic diversity in the *Candida* population, that could confer an adaptive advantage on the organism. Incidentally, these polymorphic transitions caution us that reliance on metagenomic and quantitative approaches to study the gut microbiota may not be adequately reflective of significant, populations and sub-populations that arise transiently by random and/or adaptive mechanisms.

Orthologs of the various MAPK pathway genes and also of the factors involved in the ESR have been discovered in *Candida* spp. *C. albicans* is thought to have diverged from *S. cerevisiae* more than 200 million years ago (Kurtzman and Piškur, 2006) The *C. albicans* ESR (CaESR) is not as extensive in genetic terms as in *S. cerevisiae*. Only a small number of genes are involved in CaESR (~24 upregulated genes and ~37 downregulated genes) (Enjalbert et al., 2006; Gasch, 2007). This suggests the absence of a core environmental response/general stress response in *C. albicans* (Enjalbert et al., 2003). Later studies confirmed that CaMsn4p only weakly complements the inability of an *msn2*△*msn4*△ double mutant in *S. cerevisiae* to activate a STRE-*lacZ* reporter (STRE-Stress response element) while CaMln1p *(Candida albicans* Msn2p/Msn4p-like protein) does not complement the defect at all (Nicholls et al., 2004). The transcription factors finally activated in the CaESR have not been conclusively identified. Therefore a complete picture of activation and regulation of ESR in *C. albicans* is as yet unavailable. However, the number of genes activated/repressed during the CaESR were more in response to oxidative stress (5mM H_2_O_2_) than in response to osmotic stress (0.3 M NaCl) and heavy metal stress (0.5 mM CdSO_4_) (Enjalbert et al., 2006). Other studies have reported that components of stress signaling pathways may be important in virulence, drug tolerance, or quorum sensing, among other phenotypes. Interestingly, Msn2p homologs in entomopathogenic fungi *Beauveria bassiana* and *Metarhizium robertsii* augment virulence (Zhang et al., 2009; Liu et al., 2013). Table 1 lists some components of stress signaling known to influence other phenotypes in pathogenic fungi.

**Table 1:** **Proteins involved in osmoadaptation/stress responses as well as other phenotypes in pathogenic fungi**.

**Gene/Protein**	**Function summary**	**References**
Hog1p	Enhances virulence of Ca	Alonso-Monge et al., 1999; Zhang et al., 2009
Msn2p	ESR-related transcription factor required for full virulence in entomopathogenic fungi	Liu et al., 2013
Ste11p	Shared MAPKKK in mating response and hyperosmotic stress response, implicated in Cg virulence	Calcagno et al., 2005
Hog1p	In Cn, it antagonizes the effect of Mpk1p (a MAPK) and calcineurin, which promote cell wall integrity	Kojima et al., 2006;
	Overactivation of the HOG pathway by fludioxonil (antifungal) results in fludioxinol hypersensitivity	
	Inhibition of Hog1p in Cn is associated with increased ergosterol in the cell membrane, promoting sensitivity to ergosterol-binding antifungals	Young-Sun Bahn et al., 2012
Cta1p	Catalase gene of Cg regulated by multiple stress-related transcription factors like Msn2p, Msn4p, Skn7p, Yap1p	Cuéllar-Cruz et al., 2008
Ste12p	Transcription factor induced by pheromone and nitrogen starvation in Sc, promotes virulence in Cg	Calcagno et al., 2003
Slt2p	The cell wall integrity pathway MAPK in Cg; downstream target Rlm1p (a transcription factor) is involved in micafungin tolerance	Miyazaki et al., 2010
Chk1p	Two component signal transduction protein in Ca; ortholog of oxidative stress sensors (Mak2p and Mak3p) in *S. pombe.* Involved in quorum sensing (farnesol response)	Kruppa et al., 2004
Mln1p	Msn2p-like protein enabling long-term resistance to weak acid in Ca	Ramsdale et al., 2008

Sc, Saccharomyces cerevisiae; Ca, Candida albicans; Cg, Candida glabrata; Cn, Cryptococcus neoformans.

## Conclusions

In contrast to bacteria, a beneficial role for fungi in microbiota-human interactions has not emerged, though their role as opportunistic pathogens (cf. ecologically invasive species) has been extensively studied. The major fungal probiotic in use today, *Saccharomyces cerevisiae* var. *boulardii* (Sb), that is not indigenous to the human gut, provides some examples of potential benefits of fungal proteins for the host (Czerucka et al., 1994; Dahan et al., 2003), and can help in the maintenance and/or restoration of host-microbiota homeostasis.

The strategies employed by the members of the mycobiome to adapt to changing conditions along the length of the gut and host immune responses may depend significantly on adapting to continuously changing environmental parameters that could also serve as indicators for spatial location, microbiota composition and the physiological state of the host. Signaling pathways that respond to different stimuli are not watertight modules, but can interact in unforeseen ways to produce an integrated behavioral response (Baltanás et al., 2013). Thus, components of the osmoregulatory pathway may also participate in the process of mounting a coordinated response to environmental stiumuli.

### Conflict of interest statement

Work on osmoregulation in *S. cerevisiae* in our laboratory is supported by a grant from the Department of Biotechnology, Government of India (sanction order number BT/PR12598/PBD/26/215/2009).
